# mRNA nuclear export: how mRNA identity features distinguish functional RNAs from junk transcripts

**DOI:** 10.1080/15476286.2023.2293339

**Published:** 2023-12-13

**Authors:** Alexander F. Palazzo, Yi Qiu, Yoon Mo Kang

**Affiliations:** Department of Biochemistry, University of Toronto, Toronto, Ontario, Canada

**Keywords:** mRNA nuclear export, nuclear retention, splicing, GC-content, mRNA quality control

## Abstract

The division of the cellular space into nucleoplasm and cytoplasm promotes quality control mechanisms that prevent misprocessed mRNAs and junk RNAs from gaining access to the translational machinery. Here, we explore how properly processed mRNAs are distinguished from both misprocessed mRNAs and junk RNAs by the presence or absence of various ‘identity features’.

## How the nucleocytoplasmic division acts as a quality control system for mRNA metabolism

The eukaryotic cellular space is divided into two regions, the nucleus where precursor messenger RNA (sometimes referred to as premature mRNA and is abbreviated as pre-mRNA) is synthesized and processed to form mature mRNA, and the cytoplasm where the mature mRNA is translated into protein [1,2].

This division has several benefits. First, it allows RNA processing machinery to operate on pre-mRNAs without interference from the translation machinery and vice versa. Second, this division prevents the translation of inappropriately processed pre-mRNAs, which can be deleterious to the cell. Processed transcripts are evaluated in the nucleoplasm by quality control processes and those that are misprocessed are eliminated before they reach the translational machinery in the cytoplasm. Misprocessed transcripts include those that are spliced using suboptimal exon-intron boundaries, and those cleaved pre-maturely by the polyadenylation machinery to generate intronic polyadenylation transcripts. Third, since eukaryotic genomes are largely composed of non-functional DNA that are nevertheless transcribed, the nucleocytoplasmic division allows for quality control machinery to eliminate non-functional RNAs in the nucleus before they have the chance of encountering ribosomes [3–5]. Note that there is still some debate as to whether non-functional, or ‘junk’, RNA exists. Although some long non-coding RNAs are no-doubt functional, even then most optimistic estimates suggest that they are transcribed from no more than 2% of the human genome [6] (including introns this rises to about 10%). In addition, about 35% of the genome is transcribed into pre-mRNA, of which 2% is exonic (i.e. present in the final processed product) [7]. In contrast, greater than 80% of the genome is transcribed at some level in some cell type [8], and the majority of these loci are non-functional based on both conservation estimates and biochemical data [4,6,9–12]. Thus, despite what a few critics of junk DNA/RNA claim [13,14], most of the data in the literature support the idea that eukaryotes produce a sizable amount of junk RNA. Furthermore, as described in the next section, this view is consistent with the co-evolution of the nucleus, splicing and junk DNA.

## The evolution of the nucleocytoplasmic divide and mRNA metabolism

Over the past two decades, it has become clear that the origins of the nucleocytoplasmic divide and the expansion of mRNA metabolism likely co-evolved [1,2]. It is believed that during eukaryogenesis, at least two organisms entered an endosymbiotic relationship, the first was an alpha-proteobacteria, which in time evolved into present-day mitochondria, while the second was an archaeon that itself had acquired a substantial number of eubacterial genes and may have been the product of a prior endosymbiotic event [15]. Over time, genes from the alpha-proteobacteria were absorbed into the archaea genome to form the nuclear genome. This included not only genes that code for mitochondrial-targeted proteins but also Group II introns, which eventually evolved into our spliceosome [16–18], and likely the original introns that were present in the last eukaryote common ancestor (LECA), which appears to have been intron-rich [18,19].

What remains unclear is the exact timing of when the nucleus appeared. The nucleus is a subdomain of the eukaryotic endomembrane system (i.e. the endoplasmic reticulum), and recent analyses suggest that these membranes existed in the archeal branch of our ancestry, prior to either the appearance of a nuclear pore or the acquisition of mitochondria [20]. Despite this, it remains unclear whether the nuclear envelope and nuclear pore complex evolved prior to splicing. Regardless of the exact timeline, once the nucleocytoplasmic divide was created, it likely promoted the proliferation of introns by reducing the deleteriousness of the byproducts of splicing as described above. It also likely promoted the proliferation of intergenic DNA by reducing the deleteriousness of spurious transcription [6,9].

## mRNA nuclear export: its primary role in quality control and its co-option to regulate gene expression

**Figure 1.**
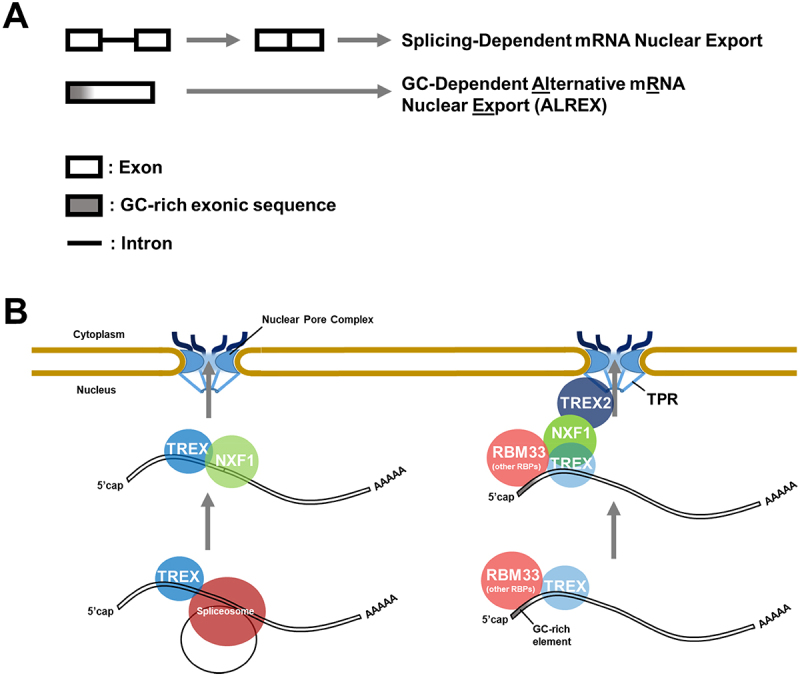
Two mRNA identity features promote the nuclear export of most mRnas. A) Schematic of the splicing-dependent and GC-dependent mRNA export pathways. B) Illustration of how each feature is recognized by trans-factors, which promote mRNA export. Note that splicing, and likely GC-rich regions, recruit the EJC to both types of mRNA (see text for details).

It is thus clear that the original, and still primary, role of mRNA export is to sort properly processed mRNAs from both misprocessed mRNAs and junk transcripts [3,5]. The mRNA export machinery accomplishes this by recognizing mRNA identity features, the most important ones being splicing [21,22] and GC-content [3,23–27] (Figure 1A). As a consequence, most mRNAs utilize one of the two main export pathways, the splicing-dependent pathway, and the GC-dependent pathway, which is sometimes referred to as the alternative mRNA export pathway, or ALREX. These same mRNA export pathways are also used by functional long non-coding RNAs that have roles in the cytoplasm. At the same time, eukaryotic cells contain a number of nuclear retention and decay pathways that recognize other features [5,28]. In some cases, mRNA identity features actively repress these nuclear retention pathways [29], while in other cases nuclear retention pathways actively suppress nuclear export pathways [30,31]. It is also likely that in many cases mRNAs that are targeted for nuclear export, simply evade nuclear RNA decay pathways [28]. Thus together, mRNA nuclear export pathways, mRNA nuclear retention pathways and RNA decay pathways coordinately act to promote the expression of functional RNAs (i.e. mRNAs and functional cytoplasmic lncRNAs) while suppressing the expression of junk transcripts [6].

Although the nucleocytoplasmic divide functions primarily to prevent unspliced pre-mRNAs, misprocessed mRNAs, and junk RNAs from entering into contact with ribosomes, the nuclear export machinery has been co-opted to regulate gene expression. Some of these include the specialized regulation of mRNA export for genes involved in cell cycle progression [32–34], innate immune activity [35], heat shock response [36] and metabolic homoeostasis [37]. In some cases, specialized export pathways recognize unique elements only under certain conditions, while in other cases particular ‘detained’ introns remain unspliced and their removal activates mRNA export [38]. The kinetics of mRNA nuclear export also helps to dampen fluctuations in protein levels that would otherwise occur due to bursts in transcription [39].

The nucleocytoplasmic divide is also used in genome defence. It can be used to limit the replication of transposable elements, especially those that have an RNA intermediate. Thus, it acts to reduce the deleteriousness of these selfish bits of DNA [40]. Although this increases the fitness of the organism, the reduction in deleteriousness also suppresses the elimination of transposable elements by purging selection.

Finally, the nucleocytoplasmic divide also plays a role in anti-viral defence in that RNAs that do not have a nuclear history tend to activate innate immune responses [40,41]. In response, many viruses try to translocate to the nucleus in order to replicate. Thus, the nucleocytoplasmic trafficking machinery is monitored by the innate immune response to detect viral invasion. In response, many viruses evolved mechanisms to counteract this by altering aspects of either the nuclear pore or nucleocytoplasmic trafficking [41].

Despite all of these additional features, the primary purpose of the nucleocytoplasmic divide is in mRNA metabolic quality control. In the next sections, we will focus on features that distinguish RNA molecules that have functionally relevant information (mostly mRNAs) from misprocessed and junk RNAs.

## Splicing: a key mRNA identity feature that promotes nuclear export

The best-characterized mRNA identity feature that promotes RNA stability and efficient nuclear export is splicing (Figure 1A). It has been widely appreciated that mRNAs from intron-containing genes are more efficiently exported than versions of the exact same mRNA produced from cDNA (i.e. intronless versions of the gene) [21,22]. How splicing and mRNA nuclear export are coupled has been well characterized and many of the molecular details of this process (Figure 1B, Table 1) are understood and reviewed elsewhere [3,5,75,76]. Upon the completion of splicing, the spliceosome helps to recruit the transcription export (TREX) complex and the exon junction complex (EJC) to the mature mRNA [42–45]. TREX in turn helps to promote efficient mRNA export by recruiting the nuclear transport receptor, composed of NXF1 and NXT1 [77–81]. The EJC has been linked to a number of mRNA metabolic steps including an increase in translation, and the removal of misprocessed mRNAs by nonsense-mediated decay [46–48]. Although the exact role of the EJC in mRNA export has remained unclear, it can bind to TREX components, such as ALYREF [46,82,83]. In the past few years, how TREX and the EJC form the core components of the messenger ribonucleoprotein (mRNP) complex has begun to be elucidated by single-particle imaging, X-ray crystallography and Cryo-EM [49,84–86].

**Table 1.** Cis-elements and features that regulate mRNA quality control.

Cis-element or feature	Associated RNAs	Trans-factors	Effect on nuclear export	Other effects
Exon-exon junctions	Spliced protein-coding mRNAs	TREX Complex, EJC [42–45]	Promotes nuclear export [21,22]	Promotes stability, translation and nonsense-mediated decay [46–48]
GC-rich 5’ ends (ALREX elements)	Protein-coding mRNAs	RBM33, SARN/THO1/CIP29 (TREX), TPR, SR proteins?, TREX2? [26, 49–51]	Promotes nuclear export [3,23–27]	Promotes stability and translation [25,52,53]
Intact 5’SS motifs	Misprocessed mRNAs (RNAs with retained introns, IPA transcripts) and lncRNAs	ZFC3H1, U1 snRNP [31,54]	Promotes nuclear retention (in conjunction with m6A) [30,54]	Promotes nuclear RNA decay, inhibits 3’ cleavage and polyadenylation [30,55–63]
m6A	Poorly spliced mRNAs, long exons, transposable element-derived RNAs	YTH domain-containing proteins [54]	Promotes nuclear retention (in conjunction with 5’SS motifs) [54]	Promotes nuclear and cytoplasmic RNA decay and heterochromatin silencing [64–67]
A-rich sequences	Exons, transposable element-derived RNAs	HUSH Complex	Unknown	Promotes nuclear RNA decay and heterochromatin silencing [68–70]
U-rich sequences	Introns	Unknown	Unknown	Relieves silencing by the HUSH Complex? [68]
dsRNA (A to I editing by ADAR)	Transposable element-derived RNAs, viral RNAs	Major I-binding RBP is unclear, Staufen (dsRNA)	Promotes nuclear retention (A to I editing) [71,72]	Promotes RNA decay (Staufen) [73,74]

Besides its role in export, exon junction density (a measure of how many splicing events give rise to a particular mRNA) is known to be one of the most important contributors to mRNA stability as assessed in a number of transcriptome-wide analyses [87–90]. In particular, deep neural networks were used to show that ORF exon density is strongly associated with increased steady-state mRNA abundance in humans [89]. More recently, a meta-analysis of transcriptome-wide mRNA decay rates (from experiments in 39 human, and 27 mouse, cell lines) also found that ORF exon junction density was the dominant feature for predicting mRNA half-life [90]. Other mRNA processing events, such as 5’ capping and polyadenylation, likely also contribute to the deposition of TREX and other nuclear export factors [91–94]; however, unlike splicing, these other processing events in isolation are insufficient to promote efficient nuclear export [5].

## GC-Content: a second mRNA identity feature that promotes nuclear export

**Figure 2.**
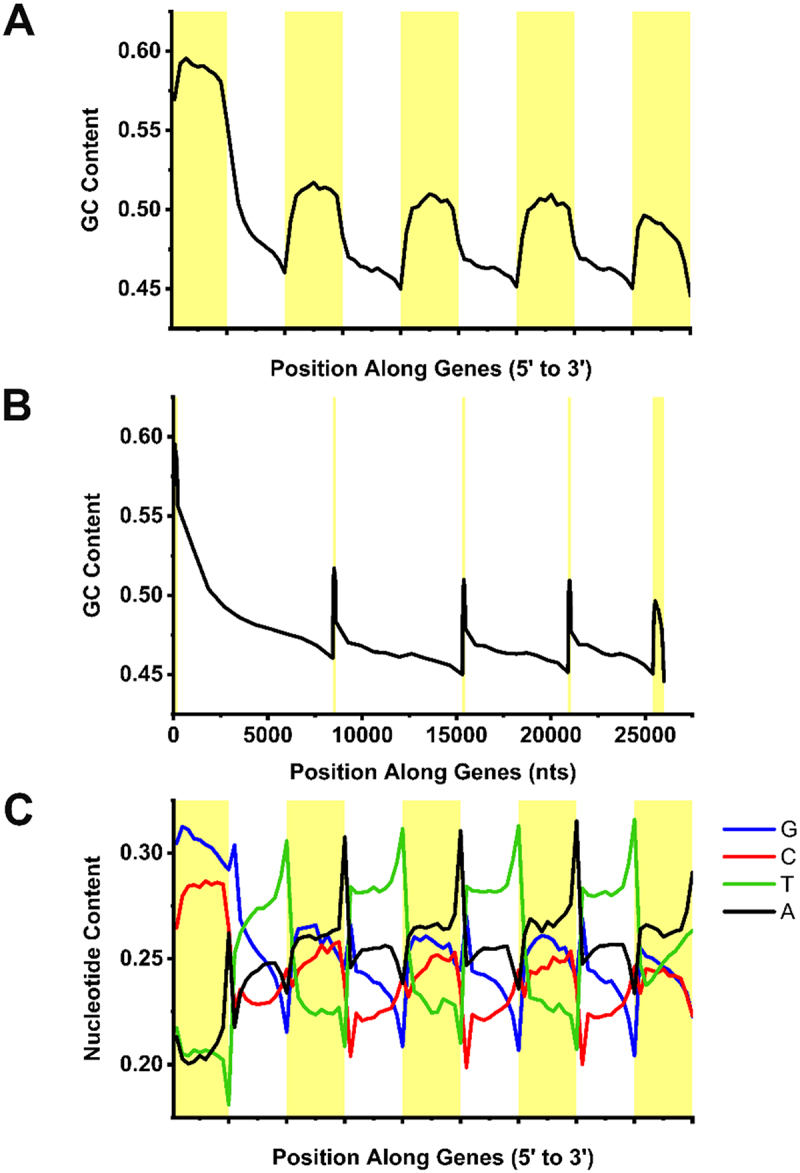
Sequence features of mRNAs. A-B) GC-content averaged over each exon (yellow) and intron (white) of all human protein-coding genes with 5 exons plotted from 5’ to 3’ ends. Note that in (A) each exon and intron metaplot was normalized, while in (B) they were adjusted to reflect the average length of each exon and intron of all genes in the dataset. C) Similar to (A) except that the average nucleotide-content of the coding strand was plotted from 5’ to 3’ ends.

The second identity feature that promotes nuclear export, is high GC-content at the 5’ end of the mRNA (Figure 1A) [27]. Intriguingly, most human protein-coding mRNAs have elevated levels of GC-content at their 5’ end regardless of whether they contain introns or not. An illustration of this is shown in Figure 2A where the average GC-content of all human genes containing five exons is plotted. Note that GC-content is highest in the first exon and decreases with every subsequent exon until it dips at the 3’ end of the gene. Introns, in contrast, are GC-poor, albeit higher than the genomic average, which is 41% GC-content for the human genome. Also, note that the exon and intron sizes in Figure 2A have all been normalized, but in reality, exons are much smaller. Indeed if we replot GC-content but take into account exon and intron sizes (Figure 2B), exons appear as GC-rich islands in a GC-poor sea.

Over 10 years ago, we proposed that mRNAs generated from intronless genes required high GC-content at their 5’ end to be efficiently exported from the nucleus to the cytoplasm [3]. In particular, we found that many signal sequence coding regions (SSCRs), which code for the peptide sequences that direct nascent polypeptides to the secretory pathway, also act as RNA elements that promote mRNA nuclear export of intronless reporter mRNAs [23]. These elements had long stretches lacking adenines, tended to be present in the first exons, and contained GC-rich elements [23,24,95]. The depletion of adenines in SSCRs is due to the enrichment between synonymous codons for those that lack adenine and between biochemically similar amino acids for those that have adenine-poor codons [23,24,95,96]. Importantly, ALREX-elements only promoted export when inserted into the 5’ end of reporter mRNAs [97]. This activity was also seen in mitochondrial targeting sequence coding regions (MSCRs) and ‘cytoplasmic accumulation RNA’ elements (CAR-Es) found in mRNAs from naturally intronless genes [95,98,99]. Again, all of these elements tended to be GC-rich and supported the idea of an alternative RNA export pathway that was sequence-dependent.

More recently, a library of mRNAs, which all coded for the exact same GFP polypeptide, but whose choice of synonymous codons were randomized, were used to identify sequence features that boosted protein expression [25]. It was determined that for intronless mRNAs, high GC-content at the 5’ end of the mRNA increased expression, and this was mostly due to increases in the efficiency of mRNA nuclear export. Interestingly, GC-content had little effect in an mRNA library that contained an intron. In parallel, another study identified elements from intronless genes that could promote the export of reporter mRNAs [26]. In agreement with the CAR-E studies, these elements were GC-rich and present near the 5’ end of certain intronless mRNAs and lncRNAs, such as *NORAD*. Furthermore, a recent pre-print in BioRxiv used machine learning to show that GC-content was one of the main drivers of high mRNA nuclear export rates [100].

The mechanism of how high GC-content at the 5’ end of RNAs promotes export has been elucidated to some extent (Figure 1B, Table 1). It was found that certain components of the TREX complex, in particular the RNA helicase UAP56 (also known as DDX39B) and its paralogue URH49 (also known as DDX39A), were required for the export or reporters with SSCRs or CAR-Es [98,99,101,102]. More recently, it was shown that depletion of the TREX component SARNP (also known as THO1 or CIP29), also had a drastic effect on the export of GC-rich mRNAs [49]. Despite this, other TREX components, such as ALYREF, do not appear to be required for GC-dependent export [51,101]. mRNAs that use the ALREX pathway also appear to require the nuclear pore basket protein TPR and use the nuclear transport receptor NXF1 to cross the nuclear pore [23,26,50,103]. Other RNA-binding factors were identified to associate with CAR-Es; however, whether these recognize GC-rich RNA and promote export remained unclear [99,104]. A recent CRISPR screen for factors that promote the nuclear export of the *NORAD* lncRNA identified RBM33 as being required for the export of intronless GC-rich mRNAs and lncRNAs [51]. This RNA binding protein not only recognizes GC-rich elements but also directly interacts with TREX components, including UAP56, ALYREF, and the nuclear transport receptor NXF1. Another complex, TREX-2, likely acts in the GC-dependent pathway as it functions with TPR [103,105]. Although many nuclear export factors are shared between the splicing and GC-dependent pathway, it appears that the depletion of any given factor tends to have a greater impact on one pathway over the other. For example, depletion of TREX components tends to have greater effects on the splicing-dependent pathway, while depletion of NXF1 and TPR has greater effects on the GC-dependent pathway [26,50,103]. These trends may be the result of competition by various mRNAs for the remaining export factors when any given component is removed.

Despite all these advances, it remains unclear how at the molecular level GC-rich regions are recognized. Despite the fact that RNA hybridization is energetically favoured in GC-rich RNA, human 5’UTRs, where most of the GC-rich sequence in mRNAs is concentrated, are not enriched for secondary structures in comparison to ORFs and 3’UTRs as assessed by chemical probes [106] and computational modelling [107]. Despite this, there appears to be a slight increase in RNA structures just upstream of the start codon and a depletion in RNA structures at the start codon [106]. Thus, it remains possible that GC-rich regions form structures that are recognized by particular RNA binding proteins. RNA structural features in pre-miRNAs and tRNAs are recognized by their nuclear transport receptors [108,109], and this may be equally true for GC-rich mRNA, but this needs further investigation.

GC-content likely impacts other aspects of mRNA biology. After exon-density, high GC-content in the 5’ UTR is the most strongly associated with increased steady-state mRNA abundance in both the human and mouse [89]. There is also some evidence that high GC-content at the 5’ end may also enhance an mRNA’s translation efficiency [25,52,96], and this may require interactions with RanBP2/Nup358, a component of the cytoplasmic filaments of the nuclear pore complex [52,110]. Interestingly, it had been observed that a subset of EJCs bind to non-canonical sites beyond simply exon–exon junctions [82]. Some of these sites were exclusively present in the first exon of spliced mRNAs and were enriched in the exact same GC-rich motif present in SSCRs [95], suggesting that the EJC may boost the translation of certain GC-rich mRNAs. GC-content may also directly enhance the efficiency of translation elongation. When synonymous human codons are compared, common codons tend to be GC-rich, and thus may be associated with higher rates of translation elongation [111]. GC-rich ORFs also protect mRNAs against cytoplasmic decay [53,111,112]. Despite all these findings, it has been found that selection between codons for translation optimality in humans is weak and the codon distribution in human protein-coding genes is mostly mediated by non-adaptive evolutionary processes, such as GC-biased gene conversion, which elevates local GC-content [113]. Indeed, it is likely that non-adaptive forces, such as GC-biased gene conversion and mutational bias, act in conjunction with adaptive forces to maintain elevated GC-content at the 5’ end of most protein coding genes [27,114].

In summary, it has become clear that GC-content is a major determinant of mRNA nuclear export and stability in mammalian cells and that these likely access particular proteins dedicated to this pathway, such as RBM33, and other proteins that are also recruited to spliced mRNAs, such as UAP56, SARNP and NXF1.

## Beyond GC-content: other nucleotide-level features of protein-coding genes

Although the GC-content of protein-coding exons has been well documented, there are other features of genes that have received less attention. For example, in human protein-coding genes the nucleotide content differs substantially between the coding and template strands and this varies along the gene length. This strand asymmetry can be visualized by plotting the individual nucleotide content on the coding strand and can be clearly seen in the collection of human protein-coding genes with five exons (Figure 2C). Some notable trends include a skew towards G and away from C within the first exon (and to a lesser extent in all other exons); a large skew towards A and away from T in internal exons; and extreme strand asymmetry within introns, especially towards T and away from A. This last asymmetry is all the more remarkable when one considers that introns are under a minimal amount of selection and that they are on average at least an order of magnitude longer than exons.

How do these patterns relate to our general understanding of mRNA metabolism? Note that the nucleotide content along the coding strand, which is shown in Figure 2C, matches the nucleotide content along the pre-mRNA. It has been observed that high GC-content in exons and low GC-content in introns may help promote proper splicing or influence how the pre-mRNA is spliced [115–117]. It is possible that other features, such as high T-content in intronic sequences, could be used to identify introns, which have correspondingly high U-content within pre-mRNAs. Indeed, it has been recently observed that U- and A-content are major determinants of how RNA derived from certain transposable elements are identified by the Human Silencing Hub (HUSH) complex (Table 1) [68,69]. It was found that A-rich nascent transcripts that are derived from LINE1 transposable elements can recruit HUSH complex, which in turn targets the RNA for decay and modifies chromatin to enforce genomic silencing. Interestingly, HUSH-mediated RNA decay and silencing can be overridden by inserting an intron into the transcribed region [68]. This likely explains why the HUSH complex does not silence most protein-coding genes, despite the fact that their internal exons are relatively A-rich (Figure 2C). Importantly, the ability of introns to suppress HUSH was not due to the recruitment of the spliceosome as introns that lacked splice site motifs still evaded HUSH silencing [68]. Since intron sequences are T-rich (Figure 2C), and the reverse complement of normally silenced transposable elements (which would also be T-rich) was also found to evade HUSH silencing, it is possible that T- and A-content are nucleotide features that can be used to distinguish protein-coding genes (whose pre-mRNA would be U-rich) from certain transposable elements (which would be transcribed into A-rich RNAs). In line with this, HUSH complex is recruited to mRNAs from intronless genes [68] and to long exons [70], which would represent long tracks of A-rich sequence, a characteristic of most exons in protein coding genes (Figure 2C).

Beyond the biases in T/U- and A-content between introns and exons, it is possible that other subtle biases in the nucleotide content of transcripts may affect RNA metabolism. For example, human genomes are depleted in CpGs due to mutational decay [118] and CpG-rich RNAs appear to be targeted for destruction and this may play a role in anti-viral defene [119].

Nucleotide trends in human genes are not widely appreciated by most molecular biologists, and their effects on RNA metabolism have not been extensively studied. This is fertile ground for future research.

## Intact splicing sequences: signals for quality control

Inevitably, splicing errors result in the failure to remove sequences from the transcripts that normally activate splicing. This includes the 5’ Splice Site (5’SS), which demarcates the boundary between the exon and an intron, the 3’ Splice Site (3’SS), which demarcates the boundary between the intron and the exon, and the branchpoint adenine, which is used to form the lariat structure during splicing. The most typical types of errors are due to splicing failure, the use of cryptic alternative splice sites, and the use of cryptic 3’ cleavage/polyadenylation sites (PASs) in introns. In many cases, this results in the presence of intact splicing signals that are typically removed during proper intron removal.

**Figure 3.**
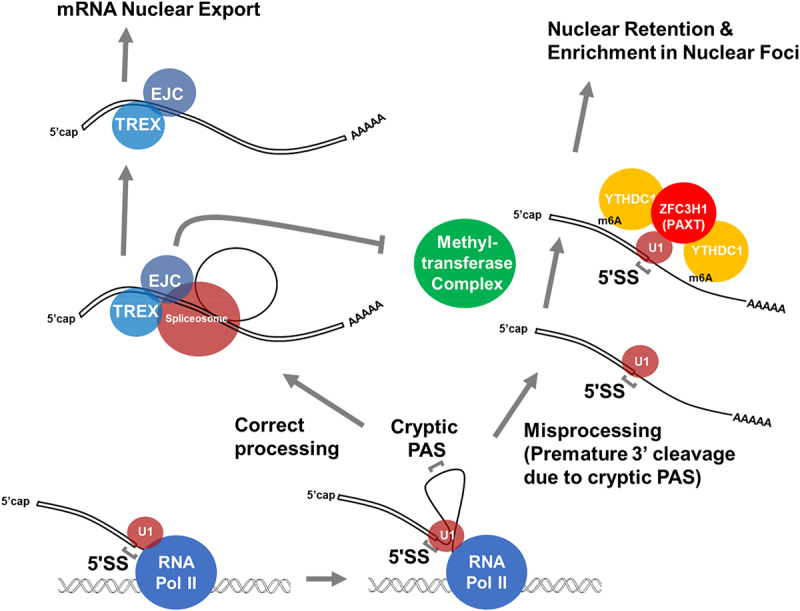
Misprocessing results in the preservation of splicing signals, which promote nuclear retention. Properly processed mRnas are compared to misprocessed mRnas that generate IPA transcripts. Note that the IPA transcript contains both an intact 5’SS due to the failure of splicing, and m6A modifications, due to the lack of deposited EJCs, which normally inhibits m6A modifications around the splice site. This could be due to the EJC sterically preventing the methylatransferase from accessing the mRNA (as depicted in the figure) or by the recruitment of demethylases such as ALKBH5.

It has been known for quite some time that the presence of intact 5’SS motifs in an mRNA triggers the inhibition of both 3’ cleavage and polyadenylation [55–60]. Indeed, when the 5’SS emerges from RNA Polymerase II it directly recruits the U1 snRNP which suppresses the activity of PASs to prevent the premature truncation of newly made transcripts [61–63]. U1 snRNP directly interacts with RNA Polymerase II and this may sterically inhibit the recruitment of the 3’cleavage/polyadenylation machinery [120]. The inhibition of premature cleavage by 5’SS motifs plays a critical role in establishing the ‘U1-PAS’ axis and preventing the expression of upstream antisense transcripts from promoters, which have a tendency of activating transcription bidirectionally [63]. However, even in the presence of a 5’SS motif, certain cryptic 3’ cleavage/polyadenylation signals may be strong enough to be used at a certain frequency to generate intronic polyadenylated (IPA) transcripts (Figure 3). In these cases, the intact 5’SS in these IPA transcripts triggers their nuclear retention and decay [30].

The molecular mechanism by which intact 5’SS promotes nuclear retention and decay is currently being elucidated (Figure 3, Table 1). This activity requires U1 snRNP and ZFC3H1, a zinc finger containing protein [31]. ZFC3H1 is part of the Poly(A) Exosome Targeting (PAXT) complex which comprises MTR4 (an RNA helicase that targets RNAs to the nuclear exosome), PABPN1 (the nuclear poly(A) binding protein) and several other components [121–124]. This complex has been implicated in the degradation of many types of RNAs by targeting them to the nuclear exosome, the main RNase in the nucleus [121]; however, it appears that certain PAXT components, like ZFC3H1, also act to prevent nuclear export of RNAs that escape degradation [31]. Although MTR4 is not required for the nuclear retention of RNAs with intact 5’SS motifs, it may inhibit the nuclear export of other RNAs by preventing the recruitment of ALYREF, a component of TREX [125]. As for PABPN1, it likely contributes to the nuclear retention of RNAs with intact 5’SS motifs by binding to the poly(A)-tail, however this activity is hard to detect as PABPN1 also promotes RNA nuclear export [94] and these two activities may cancel each other out [31].

The PAXT complex is conserved in eukaryotes, and in fission yeast (*S. pombe*) the equivalent Mtl1-Red1 Core (MTREC) complex is responsible for the nuclear retention and degradation of unspliced mRNAs, transposable element-derived mRNAs, and certain unstable non-coding RNAs [126–128]. Note that Mtl1 and Red1 are the *S. pombe* homologs of MTR4 and ZFC3H1, respectively. In both yeast and humans, ZFC3H1 binds to, and works in concert with, YTH-domain containing proteins to promote the nuclear retention of its substrate transcripts [54,126]. Although human YTH-domain containing proteins (YTHDC1 and YTHDC2) bind to *N*-6-methyladenosine (m6A) RNA modifications, in *S. pombe* the homolog (Mmi1) binds to a specific sequence motif (determinant of selective removal, or DRS) and not m6A [129]. Mmi1 also recruits 3’ cleavage machinery to nascent transcripts, and likely activates premature cleavage and decay of certain mRNAs and non-coding RNAs [130].

In both humans and fission yeast, RNAs that are retained by PAXT accumulate in nuclear foci. In *S*. *pombe*, these foci contain Red1, Mmi1 and RNA substrates [126,128,131]. In humans, RNAs with intact 5’SS motifs are first directed to nuclear speckles [30,31]. Since these structures have been implicated in post-transcriptional splicing [132], this initial targeting may help to complete the removal of introns with weak signals. However, in the case of IPA transcripts, splicing cannot be completed as they lack a branch point and a 3’SS, and these RNAs are transferred to adjacent foci enriched in YTHDC1 [54]. It is likely that these structures also form in cells when there is a general increase in m6A-enriched mRNAs [133], or when PAXT-substrates accumulate due to inhibition of the exosome [124,134]. Both YTH proteins and ZFC3H1 have intrinsically disordered regions that can form biomolecular condensates in vitro and may form the matrix of these foci [124,135,136].

Although the nuclear retention of RNAs with 5’SS likely evolved to prevent the export of misprocessed mRNAs, this system was likely co-opted by certain nuclear lncRNAs to ensure their proper localization to the nucleus [137,138]. Unlike mRNAs, lncRNAs are not depleted of 5’SS motifs in their terminal exon [30]. Furthermore, many lncRNAs are poorly spliced [139–142], and the degree to which any lncRNA is nuclear is largely dependent on whether they contain a poorly spliced intron [143], which likely triggers nuclear retention through the presence of intact 5’SS motifs. It has also been observed that lncRNA introns are often spliced using a variety of nearby 5’SS and 3’SS motifs, often leaving behind intact splice signals in the final product [142].

It is important to recognize that many annotated lncRNAs may actually be non-functional transcripts whose deleteriousness is blunted by the fact that they are retained in the nucleus and degraded [6]. Due to their reduced deleteriousness, genomic regions that produce non-functional transcripts are not effectively eliminated by natural selection, and thus we expect to see these accumulate in genomes that are under weak selection regimes, like in most multicellular eukaryotes [4]. This may be enhanced by the proliferation of transposable elements which contain promoter-like sequences that promote the transcription of intergenic regions [12,144]. Indeed, a recent study indicated that the presence of intact 5’SS motifs may help to reduce the deleteriousness of transcripts from genomic loci that eventually evolve into *de novo* lncRNA genes [145]. By triggering the decay of these intermediates and preventing them from being exported, RNA quality control lowers their potential deleteriousness and allows the loci to explore sequence space for extended periods of time [12]. Despite this, most of the available data suggest that the vast majority of these intermediates eventually lose their ability to be transcribed due to mutational decay, and thus only a vanishing small minority eventually evolve into new non-coding genes.

It is likely that other features of introns, like intact 3’SS motifs, inhibit nuclear mRNA export by recruiting a subset of spliceosome components [146–148]. How these other cis-elements promote nuclear retention will surely be the topic of future investigations.

## m6A: another layer of quality control

The *N*-6 methylation of adenine in mRNA has been a topic of intense study, although its exact role in mRNA nuclear export has been unclear due to conflicting findings. m6A is the most prevalent mRNA modification and is nonuniformly distributed across transcripts. It is enriched in the 3’ end, surrounding the stop codon, in the 3’ UTR and within long internal exons [149,150]. Recent studies have indicated that the m6A modification is excluded around splice sites by the action of the EJC [151–153] (Figure 3). It remains unclear whether the EJC sterically inhibits the m6A methylase complex or promotes m6A removal by recruiting demethylases such as ALKBH5, which has been reported to associate with several components of the EJC [154]. No matter how splicing affects m6A deposition, it is clear that an absence of m6A indicates that a particular transcript is well spliced, and the paucity of this modification, especially within the 5’ UTR and ORF, may act as an additional mRNA identity element.

Initial studies indicated that m6A methylation may promote mRNA nuclear export [155–158], however more recent studies have found that this modification tends to repress export and promote RNA decay. In particular, reducing m6A methylation by depleting the methylase (METTL3) elevated the levels of RNAs produced from intergenic regions and transposable elements [64,156,159]. Indeed, the analysis of transcriptomics data by machine learning found that the level of m6A modification of an mRNA correlates with slower nuclear mRNA export rates [100].

In mammals, m6A and m6A-binding proteins are required for the suppression of RNAs generated from transposable elements and unstable ncRNAs [64]. Other studies have shown that m6A promotes mRNA decay in the cytoplasm [65–67]. This may represent a fail-safe mechanism to destroy non-functional RNAs that are poorly spliced, and this may have been co-opted to also target the decay of certain mRNAs. m6A likely has drastic effects on RNA metabolism in the nucleus. It has been linked to the sequestration of *myc* mRNA into nuclear foci [133], and as described in the previous section, we found that the m6A modification was required for the nuclear retention of mis-spliced mRNAs [54] (Figure 3). Other groups have found that YTHDC1 interacts with components of the nuclear exosome targeting (NEXT) complex to target non-coding RNAs for decay [64]. The NEXT and PAXT complexes both share MTR4, although the former acts on RNAs before they are processed while the later acts on transcripts after they are polyadenylated [121,160]. The NEXT complex also acts with HUSH to silence certain transposable elements [161]. There are likely other connections between m6A and mRNA metabolism. For example, it has been recently observed that the mRNA nuclear export, ZC3H14 or dNab2, may actively suppress m6A deposition [162], and this could in theory further promote nuclear export.

Overall, the evidence seems quite clear that m6A acts as a layer of quality control to modify poorly processed RNAs, thus marking them as likely non-functional and thus promoting their nuclear retention and destruction.

## Adenine to inosine: a quality control mechanism to retain viral RNAs

The last feature that we will discuss is the nuclear retention of double-stranded RNAs (dsRNA, see Table 1). Many RNA viruses must transiently exist as dsRNAs to replicate their genome. These dsRNAs are recognized in the cytoplasm by a host of antiviral sensors that trigger innate immune response pathways [41]. dsRNAs can also be detected in the nucleus by RNA specific adenosine deaminase (ADAR), which converts adenines in the double strand to inosines [163], which in turn promotes nuclear retention [71,72]. As with other nuclear retention pathways, endogenous RNA substrates for ADAR tend to be transcribed from transposable elements [164–168]. In many cases, these endogenous transcripts contain two transposable element-derived sequences that are in reverse orientation from each other, which pair up to form a segment of dsRNA. Other quality control pathways, such as staufen mediated decay (SMD), may also promote the decay of endogenous transcripts that contain dcRNA regions [73,74]. Like other quality control pathways, ADAR-catalysed nuclear retention has been co-opted to regulate the expression of certain mRNAs [169].

As is the case with m6A, inosine-containing RNAs accumulate in nuclear foci. In the case of inosine-containing RNAs, these are paraspeckles [170,171]. Indeed, cells that lack paraspeckles do not have robust nuclear retention of double stranded RNA [171]. The level of ADAR activity may be further regulated by several different RNA binding proteins, and these may also play a role in the regulation of mRNA nuclear retention [172].

## Conclusion

Over the past few decades, the field has begun to understand how functionally processed mRNAs are distinguished from misprocessed and spurious transcripts. The field has also dissected how the major molecular machineries that are involved in mRNA nuclear export and retention act to distinguish these two classes of RNAs. There are, however, other features in RNA transcripts which are likely evaluated. We are only beginning to understand what these are and how they are regulated by quality control machineries. Some of these, including motifs [173] and nucleotide modifications [174], have not been confirmed by unbiased whole transcriptome analyses and await independent verification. In addition, it is clear that a major target of nuclear retention and decay are transposable element-derived RNAs, which may be recognized by particular motifs [175]. There are also other less defined nuclear retention elements [29,176,177] which will require further investigation to elucidate how they work at the molecular level.
